# Free and universal access to primary healthcare in Mongolia: the service availability and readiness assessment

**DOI:** 10.1186/s12913-019-3932-5

**Published:** 2019-02-20

**Authors:** Altantuya Jigjidsuren, Tumurbat Byambaa, Enkhjargal Altangerel, Suvd Batbaatar, Yu Mon Saw, Tetsuyoshi Kariya, Eiko Yamamoto, Nobuyuki Hamajima

**Affiliations:** 1 Department of Healthcare Administration, Nagoya University Graduate School of Medicine, 65 Tsurumai-cho, Showa-ku, Nagoya, 466-8550 Japan; 2 Asian Development Bank, Mongolia Resident Mission, Ulaanbaatar, Mongolia; 3 University of Finance and Economics, Ulaanbaatar, Mongolia; 4 National Public Health Institute, Ulaanbaatar, Mongolia; 5 Nagoya University Asian Satellite Campuses Institute, Nagoya, Japan

**Keywords:** Healthcare facility assessment, Service availability, Service readiness, Primary healthcare, Family health center, Universal access, Mongolia

## Abstract

**Background:**

The government of Mongolia mandates free access to primary healthcare (PHC) for its citizens. However, no evidence is available on the physical presence of PHC services within health facilities. Thus, the present study assessed the capacity of health facilities to provide basic services, at minimum standards, using a World Health Organization (WHO) standardized assessment tool.

**Methods:**

The service availability and readiness assessment (SARA) tool was used, which comprised a set of indicators for defining whether a health facility meets the required conditions for providing basic or specific services. The study examined all 146 health facilities in Chingeltei and Khan-Uul districts of Ulaanbaatar city, including private and public hospitals, family health centers (FHCs), outpatient clinics, and sanatoriums. The assessment questionnaire was modified to the country context, and data were collected through interviews and direct observations. Data were analyzed using SPSS 21.0, and relevant nonparametric tests were used to compare median parameters.

**Results:**

A general service readiness index, or the capacity of health facilities to provide basic services at minimum standards, was 44.1% overall and 36.3, 61.5, and 62.4% for private clinics, FHCs, and hospitals, respectively. Major deficiencies were found in diagnostic capacity, supply of essential medicines, and availability of basic equipment; the mean scores for general service readiness was 13.9, 14.5 and 47.2%, respectively. Availability of selected PHC services was 19.8%. FHCs were evaluated as best capable (69.5%) to provide PHC among all health facilities reviewed (*p* < 0.001). Contribution of private clinics and sanatoriums to PHC service provisions were minimal (4.1 and 0.5%, respectively). Service-specific readiness among FHCs for family planning services was 44.0%, routine immunization was 83.6%, antenatal care was 56.5%, preventive and curative care for children was 44.5%, adolescent health services was 74.2%, tuberculosis services was 53.4%, HIV and STI services was 52.2%, and non-communicable disease services was 51.7%.

**Conclusions:**

Universal access to PHC is stipulated throughout various policies in Mongolia; however, the present results revealed that availability of PHC services within health facilities is very low. FHCs contribute most to providing PHC, but readiness is mostly hampered by a lack of diagnostic capacity and essential medicines.

## Background

By adopting the Millennium Development Goals in 2000 and the Sustainable Development Goals in 2015 under the leadership of the United Nations, global governments have committed to ensure that everyone has access to affordable and quality healthcare [1]. Priority for service coverage under this universal health coverage plan is given to primary healthcare services based on general agreement that such services are an important prerequisite of an effective healthcare system [2, 3].

After the collapse of the socialist state system in 1990, Mongolia enacted political and economic reforms so as to move toward a democratic system with a neoliberal economy. A Semashko-style centralized and hierarchical healthcare system, which was established during the socialist regime, played a significant role in improving general health status, especially among rural residents. A strong network of soum hospitals (later renamed soum health centers: SHCs), the sole healthcare provider in rural soums (smallest administrative unit in a Mongolian province) and with a referral level at the aimag (province) level, general hospitals deliver a comprehensive set of primary and secondary healthcare provisions in rural provinces. Rural healthcare is highly resource-intensive; thus, ensuring access to health services is vital in a country with vast rural territory and a very low population density. Within urban cities, healthcare is provided through polyclinics, district hospitals, and tertiary level hospitals and specialized centers. Urban healthcare is mostly reliant on curative services, thus highly inefficient. The first two major government reforms initiated in the early 1990s were the mobilization of additional financial resources through establishing a health insurance system (i.e., in order to overcome financial shortfalls due to withdrawal from Soviet social assistance) and shifting priorities toward PHC while encouraging participation from the private sector in terms of service delivery with the aim of increasing efficiency. With the support of international development organizations, particularly the Asian Development Bank (ADB), the Mongolian government has established family group practices (FGP), which are groups of primary care physicians that provide PHC services in Ulaanbaatar (the Mongolian capital), province centers, and other cities. FGPs were envisaged as private entities under contract with local governments and financed from state budgets based on the number of registrants. The idea that “competition” among FGPs would emphasize increased quality of care and higher funding was implemented so as to encourage doctors to provide better care and services. The new system has been shaped on principles of equity and social justice, where a package of “essential” healthcare services is provided, free of charge, to everyone at the primary level, and “complimentary” health services are covered by the social health insurance system [4]. A need to reform PHC in urban cities was also urged by increasing rural to urban migration, which contributed to increases in the “urban poor” population [5], along with health inequalities due to a lack of access to basic healthcare [6].

Currently, provisions for universal, equally accessible, quality, and free-of-charge PHC is stipulated within Mongolian law [7]. The essential service package has been continuously expanded and currently includes healthcare for neonates, children and adolescents, women of reproductive age, elder adults, communicable and non-communicable diseases, emergency care, nursing, and public health services [8]. Within Mongolian cities, FGPs (later renamed family health centers; FHCs) provide PHC in outpatient settings through ambulatory counseling, daycare, and outreach services. The SHC in rural soums, owing to geographical distances and a vast catchment area, also operate 10–15 beds for inpatient care, basic surgery, and delivery services. Currently there are 549 PHC providers, including 218 FHCs in Ulaanbaatar and other cities, as well as 331 SHCs in rural provinces. As of 2018, PHC providers adsorb 18.6% of total government health expenditures and employ 5814 staff, including medical doctors, nurses, and other professional and support staff, which comprise 11.5% of the total healthcare workforce. FHCs and SHCs perform 48.9% of all outpatient consultations and SHCs treat 15.4% of all inpatients in Mongolia.

The role of SHCs in providing PHC in remote rural areas is widely accepted and recognized, which is not the case for FHCs. FHCs are often ignored, as city residents can easily bypass FHCs and directly access a broader range of services available in public and private hospitals. National and international experts have noted that since initial implementation, the FGP model has gradually eroded and been mishandled to the point that if not addressed properly, the whole initiative is in danger of failing completely [9]. Client satisfaction surveys, conducted under different projects by different stakeholders, often report low satisfaction among users in terms of quality and availability of FHC services, as well as staff attitudes and communication. Therefore, the present study examined the (i) availability of PHC services among FHCs in Ulaanbaatar and FHC readiness to provide those services; (ii) availability of PHC services in other healthcare facilities, including hospitals, clinics, and sanatoriums; and (iii) discuss the reasons that PHC provisions are hampered when they should be easily accessible and universal in Mongolia.

## Methods

### Study design

The present descriptive cross-sectional study was based on the service availability and readiness assessment survey conducted in Chingeltei and Khan-Uul districts of Ulaanbaatar. This was done in order to establish baseline data for a new project that will be implemented with financial and technical support from the ADB. SARA is a tool developed by WHO for generating a set of indicators that provide information regarding whether or not a facility meets the required conditions for supporting provisions of basic or specific services with a reliable level of quality [10]. Such information can be used in various ways, mostly in planning and managing health systems, planning and monitoring the progress of health interventions, and, in this case, to monitor outputs of an investment program. The survey deployed 201 tracer indicators under three main focus areas: general service availability, general service readiness, and service-specific readiness. A summary of indicators and definitions are presented in Table 1.

**Table 1:** Summary of definitions and indicators used in service availability and readiness assessment survey

	Definitions	Parameters/Indicators
General service availability	Physical presence of the delivery of services and encompasses health infrastructure, core health personnel and aspects of service utilization	
	Compared to target or benchmark: (i) Health infrastructure	- Number of health facilities per 10,000 population - Number of inpatient beds per 10,000 population
	(ii) Health workforce	- Number of health workers per 10,000 population - Nurse to doctor ratio
	(iii) Service utilization	- Hospital discharges per 100 population per year - Outpatient visits per capita per year
General service readiness	Overall capacity of health facility to provide general health services at minimum standards. Defined as availability of components required to provide services, such as basic amenities, basic equipment, standard precautions for infection prevention, diagnostic capacity and essential medicines.	
	Mean of average scores in five domains: (i) Facility	Mean availability of 7 items (%): power source, water source, sanitation, rooms with auditory and visual privacy, communication equipment, access to internet, and emergency transport.
	(ii) Basic equipment	Mean availability of 6 items (%): adult scale, child and infant scale, thermometer, stethoscope, blood pressure apparatus, and light source
	(iii) Infection prevention	Mean availability of 11 items (%): guidelines for infection prevention, eye protection, gowns, masks, latex gloves, disposable syringes, disinfectant and alcohol-based hand rub, appropriate storage of infectious waste, appropriate storage of sharp waste, safe disposal of sharps, sterilization equipment
	(iv) Diagnostics	Mean availability of 10 items (%): hemoglobin, blood glucose, urine protein, urine glucose, alanine-aminotransferase and creatinine, HIV, syphilis, pregnancy test, TB microscopy, general microscopy
	(v) Essential medicines	Mean availability of 13 items (%): diazepam 5 mg, amitriptyline 25 mg, paracetamol suspension 250 mg/5 ml, omeprazole 20 mg, glibenclamide 5 mg, captopril 25 mg, simvastatin 20 mg, atenolol 50 mg, salbutamol 0.1 mg, co-trimoxazole 8 + 40 mg/ml, amoxicillin 50 mg, ciprofloxacin 500 mg, ceftriaxone 1 g injection.
Service-specific readiness	Ability of health facility to offer a specific service and the capacity to provide that service. Measured through consideration of tracer items, including trained staff, guidelines, equipment, diagnostic capacity and medicines and commodities.	
	(i) Family planning (ii) Antenatal care (iii) Routine immunization (iv) Preventive and curative care for children under-five years old (v) Adolescent health services (vi) Tuberculosis services (vii) HIV and STI services (viii) Diagnostic and management of NCDs (ix) Basic obstetric and newborn care (x) Basic surgical services (xi) Blood transfusion	For each services the readiness score is computed as the mean availability of service-specific items in four domains: (i) Trained staff and up-do-date standards, (ii) Functioning equipment, (iii) Diagnostics capacity (iv) Availability of medicines and commodities.

### Sample size and sampling units

The survey was conducted in two districts, Chingeltei and Khan-Uul in Ulaanbaatar. The districts were purposely selected by the government as the target districts for the ADB financed project. Both districts have similar population sizes, though their socio-economic characteristics are a bit different. Living conditions are more favorable in Khan-Uul, where 54.5% of households live in comfortable apartments and houses, while in Chingeltei, 79.8% of households reside in traditional “ger” housing, with no sanitation, water supply, or centralized heating system [11]. Multidimensional poverty, which counts economic and non-economic dimensions, was higher in Chingeltei according to a World Bank report [6].

The assessment was carried out in all public and private health facilities within these two districts. The facilities included (i) district health centers, (ii) district hospitals, (iii) private hospitals, (iv) hospitals for special civil servants, (v) sanatoriums or private institutions that provide rehabilitation services using alternate/traditional medicine; (vi) private outpatient clinics that offer specialist services in one particular discipline (most common are dental, genecology, ophthalmology, and pediatrics); and (vii) FHCs managed by private entities, namely partnerships of family practitioners based on a tripartite service contract with the local government and city health department. A total of 170 health facilities are contained within the two districts; however, the present study covered 146 facilities (85.9%), leaving aside one special health facility for prisoners, 16 private outpatient clinics, six sanatoriums that were closed or were not located at the documented address, and an affiliate of one FHC that was only open during the winter.

### Data collection

The SARA questionnaire was slightly adjusted to the country context by disregarding questions related to malaria services and compiling questions related to human immunodeficiency virus (HIV) treatment, care, and support, as HIV is minimal in Mongolia. A pre-test questionnaire was administered, and required modifications were completed. The data collection work plan was also refined after pre-testing within two health facilities.

The assessment was conducted between May and October 2017 by the Mongolian Association of Healthcare Quality Management. Four teams consisting of four researchers were deployed for data collection. Training for data collectors was conducted to ensure accuracy and reliability of data procurement and entry. The field supervisors first communicated, face-to-face, with management of the health facility to explain the study’s purpose. After obtaining consent from management, data were collected by interviewing relevant personnel and through direct observation. Data collectors entered information in paper format and dispensed to field supervisors for review and crosschecking. Field supervisors reviewed every questionnaire and if additional clarifications were required or information was incomplete, the questionnaire was returned to the data collectors. After validation for accuracy and completeness, the data were entered into an electronic database.

### Data analyses

Data were exported and analyzed using a Statistical Packages for Social Science (SPSS) software program version 21.0 (IBM SPSS Inc.) following the SARA manual. To assess general service availability across the two districts, we created a master list of all health facilities registered, and we reviewed all health facilities that were operational at the time of assessment. All health facilities were stratified into four groups: FHCs, private clinics, sanatoriums, and hospitals. District health centers, district hospitals, private hospitals, and a hospital for special civil servants were grouped as “hospitals”. Population data were taken from national annual statistics reports, and information on service utilization, health workforce, and capacity were taken from routine national health information management system databases [12]. The indicators were expressed as a percentage score and compared with international benchmarks.

To assess general service readiness, we first calculated scores for each of five domains (amenities, basic equipment, infection prevention, diagnostic capacity, and essential medicines) based on mean availability of tracer items as a percentage within the domain. Then mean of all five domains was calculated and expressed as a general service readiness index. The same approach was used when computing the service-specific readiness score. For each of the 11 selected services, a readiness score was computed as the mean availability of service-specific items across four domains (trained staff and up-do-date standards, functioning equipment, diagnostic capacity, and availability of medicines and commodities). Readiness was calculated via a frequency and mean level of differences between groups with 95% confidence intervals (95% CI). A Kolmogorov–Smirnov test was performed to determine normality of variances. As the variances were not normally distributed, relevant nonparametric tests, such as the Mann-Whitney U and Kruskal-Wallis tests, were used to compare median parameters. Also, Chi-square tests were used to compare categorical variables.

## Results

### General availability and readiness of health services

Out of the 146 health care facilities in Chingeltei and Khan-Uul, 75.3% were private, 21.2% were government-owned and privately managed FHCs, and only 3.4% were pure public entities (Table 2). The density of core health workers (physicians, nurses, and midwifes) was 50.6 per 10,000 population in Chingeltei and 73.8 per 10,000 in Khan-Uul (the international benchmark is 23). The nurse-to-doctor ratio was 1.4 to 1 (the international benchmark is 4 to 1). Health facility density ranged from 5.1 per 10,000 population in Chingeltei to 4.1 per 10,000 in Khan-Uul (the international benchmark is 2). The number of hospital discharges significantly varied in the two districts: 6.6 per 100 population in Chingeltei and 14.1 per 100 in Khan-Uul (the international benchmark is 10). The number of outpatient visits was relatively similar in the two districts: 5.9 and 6 visits per capita per year in Chingeltei and Khan-Uul, respectfully (the international benchmark is 5).

**Table 2:** Characteristics of the health facilities and service density in two districts in Ulaanbaatar, Mongolia, 2017

Characteristics	CHD	KHD	Total	International benchmark ^a^
Population	158,014	159,465	317,479	
Number of health facilities in sample	80	66	146	
Facilities by ownership and managing authority				
Private, %	78.75	71.21	75.34	
Private management, %	18.75	24.24	21.23	
Public, %	2.50	4.55	3.42	
Facilities by level of service				
Primary level, %	18.8	24.2	21.2	
Secondary ^b^, %	68.8	60.6	65.1	
Health service density:				
No. of facilities per 10,000 population	5.1	4.1	4.6	2
Inpatient beds per 10,000 population	14.4	28.8	21.6	25
Hospital discharges per 100 population	6.6	14.1	10.4	10
Outpatient visits per person per year	5.9	6.0	5.9	5
No. of core health personnel per 10,000	50.6	73.8	61.2	23
Nurse to doctor ratio	1.3:1	1.5:1	1.4:1	4:1

^a^Source: World Health Organization; ^b^ includes clinics and excludes sanatoriums

CHD = Chingeltei district; KHD = Khan-Uul district

The general service readiness score, or overall capacity of the health facilities, in the two districts to provide basic services at minimum standards was 44.1% [95% CI: 36.1–51.9] (Table 3). Sanatoriums and private clinics scored particularly low (40.3 and 36.3%, respectively), and FHCs and general hospitals scored slightly higher (62.4 and 61.5%, respectively).

**Table 3:** Mean scores for general service readiness in two districts in Ulaanbaatar, Mongolia, according to SARA, 2017

	CHD	KHD	Total	*P* value	By facility level				
					FHCs	Clinics	Sanatoriums	Hospitals	P value
Number of facilities									
	80	66	146		31	88	20	7	
Basic amenities, 7 items (% [95% CI])									
	68.1 [56.6–76.7]	70.4 [57.8–79.4]	69.1 [61.3–76.1]	0.080	77.4 [60.2–88.6]	64.3 [54.4–73.9]	73.6 [53.8–88.8]	78.6 [48.7–97.4]	< 0.001
Basic equipment, 6 items (% [95% CI])									
	40.4 [29.9–50.9]	58.4 [47.1–70.1]	47.2 [40.7–56.7]	0.030	88.5 [71.1–94.8]	27.1 [19.1–37.4]	34.3 [18.1–56.7]	80.0 [48.7–97.5]	< 0.001
Standard precautions, 11 items (% [95% CI])									
	73.2 [63.2–82.1]	75.0 [64.2–84.5]	76.0 [66.3–80.4]	0.329	81.5 [63.7–90.8]	73.3 [63.8–81.9]	80.0 [58.4–91.9]	78.8 [48.7–97.6]	0.025
Diagnostics, 10 items (% [95% CI])									
	11.1 [6.0–20.0]	16.8 [9.6–27.4]	13.9 [9.1–20.2]	0.179	38.4 [23.7–56.2]	3.5 [1.2–9.5]	0.5 [0.0–0.2]	66.7 [35.9–91.8]	< 0.001
Medicines, 13 items (% [95% CI])									
	11.4 [6.6–19.1]	18.4 [10.7–29.1]	14.5 [9.6–20.1]	0.442	21.8 [11.4–39.8]	13.1 [7.9–22.3]	13.1 [5.2–36.1]	7.7 [2.6–51.3]	0.130
General service readiness index [95% CI]									
	40.8 [31.1–52.2]	47.8 [36.8–60.3]	44.1 [36.1–51.9]	0.021	61.5 [43.8–76.3]	36.3 [27.1–46.8]	40.3 [21.9–61.3]	62.4 [25.1–84.2]	< 0.001

CHD = Chingeltei district; FHCs = family health centers; KHD = Khan-Uul district; SARA = service availability and readiness assessment

Diagnostic capacity was very low, with only 13.9% [95% CI: 9.1–20.2] of all 10 basic diagnostic items available across all facilities. Although these basic tests should be available in most settings, only one facility scored 100.0% (Chingeltei district general hospital). Diagnostic capacity within private outpatient clinics was extremely low, where any one type of basic diagnostic test was offered in only 3 (3.5%) clinics out of 88. FHCs, in general, exhibited poor capacity (38.4%). Simple procedures, such as blood glucose tests (51.6%), urine protein tests (41.9%), and blood hemoglobin testing (6.5%), were not widely available.

The presence of 13 essential medicinal items was also low (14.5% [95% CI: 9.6–20.1]), with alarmingly low average scores for public hospitals (7.7%), private outpatient clinics (13.1%), and sanatoriums (13.1%). Essential medicines, such as those for treating infectious diseases (ceftriaxone 1 g, ciprofloxacin 500 mg, amoxicillin 500 mg, and co-trimoxazole 8 + 40 mg/ml), for managing diabetes (glibenclamide 5 mg), and for neurological disorders (amitriptyline 25 mg, and diazepam 5 mg), were not available in all hospitals reviewed. The availability of tracer medicines within FHCs was also low (21.8%).

Availability of six basic equipment items was 47.2% [95% CI: 40.7–56.7] on average, which was higher in hospitals (80.0%) and lower in sanatoriums (34.3%) and clinics (27.1%). FHC capacity was 88.5%. Very basic elements, like blood pressure measurement devices and stethoscopes, were the least likely to be missing, though not universally available. Compliance with standard protection measures for infection prevention and control should be 100% across all types of facilities. However, rates ranged from 73.0 to 81.5%, regardless of ownership and facility type.

In terms of health facility infrastructure, availability of basic amenities, such as water, a power source, sanitation, communication, Internet connections, and emergency transport, ranged between 64.3% for private outpatient clinics to 78.6% for hospitals. However, none of the private clinics, and only 16.7% of hospitals and 9.7% of FHCs, had all seven items. Emergency transport was the least available (20.5% across all facilities).

Overall, only 40.8% [95% CI: 31.1–52.2] of health facilities in Chingeltei and 47.8% [95% CI: 36.8–60.3] in Khan-Uul had the capacity to provide basic health services at minimum standards. General service readiness scores were relatively higher for hospitals (62.4%) and FHCs (61.5%). Only one third of sanatoriums (40.3%) and private clinics (36.3%) were able to comply with the required minimum standards.

### Availability of PHC

Table 4 shows the availability of specific PHC services across the health facilities surveyed. The SARA assessment revealed that availability of the 11 selected PHC services was 17.0% in Chingeltei and 23.1% in Khan-Uul. FHCs that offer all but basic obstetric and newborn care, surgery, and blood transfusion services were the highest in terms of their capacity to provide PHC among all health facilities reviewed, with a mean score of 69.5%. The next most capable were hospitals, which had a score of 56.7%, while contributions from private clinics and sanatoriums were minimal, with mean scores of 4.1 and 0.5%, respectfully. The overall density of health facilities offering PHC services was very low, below 1 facility per 10,000 population compared to a total health care facility density of 4.6 per 10,000.

**Table 4:** Availability of primary healthcare services in two districts in Ulaanbaatar, according to SARA, 2017

	CHD	KHD	Total	P value	Density per 10,000	By facility level				P value
						FHCs	Clinics	Sanatoriums	Hospitals	
Family planning services (%)										
	30.0	31.8	30.8	0.813	1.4	100.0	12.5	0.0	50.0	< 0.001
Antenatal care services (%)										
	22.5	28.8	25.3	0.385	1.2	100.0	3.4	0.0	60.0	< 0.001
Routine immunization services (%)										
	20.0	27.3	23.3	0.301	1.1	100.0	0.0	0.0	50.0	< 0.001
Preventive and curative care for children under 5 years old (%)										
	26.3	39.4	32.2	0.091	1.5	100.0	11.4	5.0	66.7	< 0.001
Adolescent health services (%)										
	26.3	36.4	30.8	0.188	1.4	100.0	12.5	0.0	50.0	< 0.001
Tuberculosis services (%)										
	21.3	25.8	23.3	0.521	1.1	96.8	1.1	0.0	33.3	< 0.001
HIV counselling and testing, and STI services (%)										
	16.3	21.2	18.5	0.442	1.0	71.0	2.3	0.0	50.0	< 0.001
NCD diagnosis and/or management (%)										
	21.3	31.8	26.0	0.148	1.2	96.8	2.3	0.0	83.3	< 0.001
Basic obstetric and newborn care (%)										
	0.0	1.5	0.7	n/a	0.1	0.0	0.0	0.0	16.6	n/a
Basic surgery (%)										
	2.5	4.5	3.4	0.826	0.2	0.0	0.0	0.0	80.0	< 0.001
Blood transfusion (%)										
	1.3	6.1	3.4	0.257	0.2	0.0	0.0	0.0	83.3	< 0.001
Specific service availability score (%)										
	17.0	23.1	19.8	0.27	0.9	69.5	4.1	0.5	56.7	< 0.001

CHD = Chingeltei district; FHCs = family health centers; HIV = Human Immunodeficiency Virus; KHD = Khan-Uul district; NCD = non-communicable disease; SARA = service availability and readiness assessment; STI = Sexual transmitted infections

### Service-specific readiness

Readiness of the health facilities offering PHC services was assessed separately in the selected 11 interventions (Table 5).

**Table 5:** Readiness of facilities offering primary healthcare services in two districts in Ulaanbaatar, Mongolia, according to SARA, 2017

	FHCs		Hospitals		Clinics		Sanatoriums		All facilities	
	SSAS	SSRS	SSAS	SSRS	SSAS	SSRS	SSAS	SSRS	SSAS	SSRS
Family planning services (% [95% CI])										
	100.0 [88.9–100.0]	44.0 [29.2–62.2]	50.0 [39.8–60.2]	54.2 [44.5–63.6]	12.5 [5.2–36.0]	27.3 [11.2–46.9]	0.0 [0.0–3.5]	0.0 [0.0–3.5]	30.8 [23.9–38.7]	34.5 [27.0–42.3]
Antenatal care services (% [95% CI])										
	100.0 [88.9–100.0]	56.5 [40.7–73.6]	60.0 [49.8–69.8]	95.8 [89.9–98.3]	3.4 [0.8–23.6]	79.2 [58.4–91.9]	0.0 [0.0–3.5]	0.0 [0.0–3.5]	25.3 [18.9–32.9]	40.2 [32.8–48.5]
Routine immunization services (% [95% CI])										
	100.0 [88.9–100.0]	83.6 [67.4–92.9]	50.0 [39.8–60.2]	76.7 [67.5–83.9]	0.0 [0.0–0.2]	0.0 [0.0–1.1]	0.0 [0.0–3.5]	0.0 [0.0–3.5]	23.3 [17.2–30.8]	79.3 [72.2–85.2]
Preventive and curative care for children under 5 years old (% [95% CI])										
	100.0 [88.9–100.0]	44.5 [29.2–62.2]	66.7 [56.7–75.9]	40.6 [31.5–50.4]	11.4 [2.8–30.1]	30.0 [14.5–51.9]	5.0 [2.6–51.3]	31.3 [23.1–40.9]	32.2 [25.1–40.1]	41.3 [33.4–49.2]
Adolescent health services (% [95% CI])										
	100.0 [88.9–100.0]	74.2 [56.7–86.3]	50.0 [39.8–60.2]	88.9 [81.3–93.7]	12.5 [5.2–36.0]	29.2 [14.5–51.9]	0.0 [0.0–3.5]	0.0 [0.0–3.5]	30.8 [23.9–38.7]	64.1 [56.3–71.7]
Tuberculosis services (% [95% CI])										
	96.8 [83.8–99.4]	53.4 [37.7–70.8]	33.3 [24.0–43.3]	100.0 [96.3–100.0]	1.1 [0.8–23.6]	91.7 [69.9–97.2]	0.0 [0.0–3.5]	0.0 [0.0–3.5]	23.3 [17.2–30.8]	56.8 [48.7–64.6]
HIV counselling and testing, and STI services (% [95% CI])										
	71.0 [53.4–83.9]	52.2 [34.8–68.0]	50.0 [39.8–60.2]	75.0 [65.7–82.5]	2.3 [0.8–23.9]	56.8 [34.2–74.2]	0.0 [0.0–3.5]	0.0 [0.0–3.5]	18.5 [13.0–25.6]	52.5 [44.7–60.7]
NCD diagnosis and/or management (% [95% CI])										
	96.8 [83.8–99.4]	51.7 [34.8–68.3]	83.3 [73.7–89.4]	41.8 [32.6–51.6]	2.3 [0.8–23.9]	25.9 [11.2–46.8]	0.0 [0.0–3.5]	0.0 [0.0–3.5]	26.0 [19.6–33.7]	39.1 [31.5–47.1]
Basic obstetric and newborn care (% [95% CI])										
	0.0 [0.0–1.1]	0.0 [0.0–1.1]	16.6 [10.6–26.2]	100.0 [96.3–100.0]	0.0 [0.0–1.6]	0.0 [0.0–1.6]	0.0 [0.0–3.5]	0.0 [0.0–3.5]	0.7 [0.1–3.7]	100.0 [97.4–100.0]
Basic surgery (% [95% CI])										
	0.0 [0.0–1.1]	0.0 [0.0–1.1]	80.0 [70.0–86.6]	91.8 [84.8–95.8]	0.0 [0.0–1.6]	0.0 [0.0–1.6]	0.0 [0.0–3.5]	0.0 [0.0–3.5]	3.4 [1.5–7.8]	91.8 [86.2–95.2]
Blood transfusion (% [95% CI])										
	0.0 [0.0–1.1]	0.0 [0.0–1.1]	83.3 [73.7–89.4]	91.9 [84.9–95.8]	0.0 [0.0–1.6]	0.0 [0.0–1.6]	0.0 [0.0–3.5]	0.0 [0.0–3.5]	3.4 [1.5–7.8]	91.9 [84.2–95.2]

FHCs = family health centers; HIV = Human Immunodeficiency Virus; NCD = non-communicable disease; SARA = service availability and readiness assessment; STI = sexual transmitted infections; SSAS = specific service availability score; SSRS = specific service readiness score

***Family planning services***, one of the key elements for maternal, child, and reproductive health, were offered in 45 facilities (30.8%) across both districts, with an overall readiness score of 34.5%. Family planning services were more available in FHCs (100.0%) than hospitals (50.0%) and private clinics (12.5%). However, readiness among FHCs regarding counseling and providing family planning tools was at 44.0% [95% CI: 29.2–62.2], mostly due to a lack of oral contraceptives, injectable contraceptives, and male condoms, as required per national standards [13, 14].

***Antenatal care services*** were provided by 37 health facilities (25.3%). National guidelines envisaged an ample set of preventive and curative activities related to ante- and postnatal care, where FHCs play a central role in screening pregnant women, regular monitoring during and after the pregnancy, and timely referrals to specialists if needed [15]. Readiness among FHCs in regard to equipment supply (100.0%) and trained staff (80.2%) was adequate; however, only 67.7% of FHCs could check hemoglobin levels in the blood and protein levels in urine; furthermore, few facilities had iron and folic acid tablets, as well as the tetanus toxoid vaccine in stock (8.6%). Overall readiness among FHCs to provide antenatal care services was at 56.5% [95% CI: 40.7–73.6]. In regard to other facilities, 60.0% of hospitals and 3.4% of private clinics (3 out of 88 clinics) offered antenatal care; however, these facilities had relatively high readiness (95.8 and 79.2%, respectfully).

***Routine immunization services*** were offered by 34 facilities (23.3%). Readiness among health facilities to offer routine immunization services was assessed by looking at the presence of vaccines (measles, diphtheria, pertussis, polio, hepatitis B, hemophilus influenzae, and TB), cold chain equipment, and trained staff and guidelines. Routine immunization services were offered in all FHCs (100.0%) and half of the hospitals (50.0%). Both FHCs and hospitals reported sufficient capacity in terms of trained staff and guidelines (92.6%). However, vaccines were not widely available (65.5%), particularly the BCG vaccine, which was available in 19.4% of FHCs and 33.0% of hospitals. Overall readiness among FHCs to provide routine immunization services was 83.6% [95% CI: 67.4–92.9], while readiness among hospitals was at 76.7%.

***Preventive and curative care for children under-five years old*** was provided by 47 facilities (32.2%). All FHCs (100.0%) offered preventive and curative services for children under-five, but service readiness was only 44.5% [95% CI: 29.2–62.2], with major deficiencies in diagnostic capacity (6.5%) and medicinal supply (18.9%). Only 2 out of 31 FHCs had the minimum required diagnostic tests (i.e., hemoglobin and parasite tests). Availability of essential medicines, such as co-trimoxazole syrup, paracetamol suspension, and me/albendazole capsules, varied from 6.5 to 12.9%. Readiness among hospitals was also scored at 40.6% due to nonexistent essential medicines and limited availability of diagnostic tests. For the same reason, readiness among 10 pediatric clinics and the one sanatorium that served children was evaluated at 30.0 and 31.3%, respectively.

***Adolescent health services*** were offered in 45 facilities (30.8%), including FHCs (100.0%), hospitals (50.0%), and private clinics (12.5%). Specific criteria used for defining service readiness, such as the presence of a facility to provide STI and reproductive health services, staff trained in adolescent health, and condom distribution, were higher in hospitals (91.7, 83.4, and 66.7%, respectively) compared to FHCs (95.2, 59.7, and 35.5%, respectively). Overall readiness among hospitals was 88.9%, readiness among FHCs was 74.2% [95% CI: 56.7–86.3], and only 29.2% of private clinics that offer adolescent health services were ready to provide services.

***Tuberculosis services*** were offered by 34 facilities (23.3%). According to national guidelines, TB services at the primary level comprise preventive screening, vaccination, diagnosis (sputum microscopy), and treatment of non-complicated and drug sensitive cases [16]. Analysis of relevant primary level questions from the SARA survey revealed that all but one FHC offered TB services (96.8%); however, FHCs mostly relied on symptom-based diagnostics due to a lack of diagnostic capacity (25.5%), and only 6.7% had all five first-line anti-TB drugs. Consequently, readiness among FHCs for TB services was rated at 53.4% [95% CI: 37.7–70.8], while readiness among hospitals was at 100%. Readiness within the one private TB clinic was 91.7%.

***HIV counseling, testing, and STI services*** were offered in 27 facilities (18.5%). The role of FHCs was limited to counseling, preventive behavior change communication, and screening pregnant women and high-risk population groups against HIV and STI with rapid tests. Suspected cases are referred to the district hospital for further diagnosis and treatment [17]. Results revealed that the weakest component was ensuring a patient’s privacy due to facility limitations (31.8%). Availability of HIV and syphilis rapid tests was 72.8%; however, drugs for STI treatment (metronidazole, ciprofloxacin, and injectable ceftriaxone) were not available within the FHCs. The overall readiness score among FHCs was 52.2% [95% CI: 34.8–68.0], 75.0% among hospitals, and 56.8% within private clinics. Out of the two private clinics specialized in STIs, only one had syphilis testing equipment and the required medicines.

Readiness among health facilities for ***diagnosis and/or management of non-communicable diseases (NCD)*** was assessed in 43 facilities (28.3%) that offered NCD services. The study selected three interventions, such as diagnosis and treatment of diabetes, cardiovascular diseases (CVD), and chronic respiratory diseases (CRD). Overall readiness across all facilities was 55.1% for diabetes services, 58.5% for CVD services, and notably lower for CRD services (18.3%). Regarding facility type, hospitals had higher readiness scores (71.5% for diabetes, 62.0% for CVDs, and 54.6% for CRDs) than private clinics (20.9, 45.8, and 25.0%, respectively). There were 3 sanatoriums that offered services for CVDs and CRDs, with readiness ranging from 66.7% (CVDs) to 100.0% (CRDs). Readiness among FHCs was 52.5% for diabetes services, 47.8% for CVDs, and 42.1% for CRDs. Across all types of facilities, readiness for CRDs was lower compared to the other two diseases, mainly due to a lack of guidelines and trained staff (20.9%), diagnostic capacity (31.5%), and the absence of medicines for treatment (10.8%). The weakest domain across all facilities for NCD diagnosis and management was availability of medicines, which ranged between 7.2 and 26.1%. Essential medicines for diabetes management, such as metformin, injectable insulin, and glibenclamide, were largely unavailable (15.8%), while the situation regarding first-hand medicines for CRDs (such as salbutamol, prednisolone, beclomethasone, hydrocortisone, epinephrine, and oxygen) were even worse (10.8%). Overall readiness for NCD diagnosis and management was 51.7% [95% CI: 34.8–68.3] for FHCs, 41.8% for hospitals, and 25.9% for private clinics.

***Basic obstetric and newborn care*** was offered in only one private hospital in Khan-Uul (Intermed Hospital), and readiness in terms of trained staff, equipment, and medicines was sufficient (100.0%, [95% CI: 95.8–100.0]).

***Basic surgical services*** were only available in five hospitals (3.4% of all health facilities). Among the index services, the most frequently provided were wound debridement, removal of foreign objects, and suturing. The overall readiness score was 91.8% [95% CI: 84.5–96.1].

***Blood transfusions*** were also offered in five hospitals (3.4%). All had staff trained on safe practices, guidelines, blood typing, cross matching testing, and used standard equipment. Overall readiness was 91.9% [95% CI: 84.5–96.1].

## Discussion

The present study revealed serious limitations with ensuring universal access to basic health services in Mongolia. The physical presence of a health infrastructure in the two districts sampled indicated that the number of health facilities and health personnel is 2–3 times higher compared to international benchmarks; however, only 44.1% of health facilities had the capacity to provide basic health services at minimum standards. Hospitals and FHCs were more likely to meet minimum standards, but most private clinics and sanatoriums did not. The overall general service readiness score was lower compared to other low-income countries [18–22]. Additionally, the availability of PHC services across all health facilities tested was as low as 19.8%. The important services, such as family planning, routine immunization, antenatal care, preventive and curative care for children under-five years old, and non-communicable and communicable disease diagnosis and management, were only accessible in FHCs. Half of the hospitals offered PHC services but were only accessible for those who could pay. Private clinics and sanatoriums, which comprised 74% of the healthcare facilities across the two districts, did not offer any of these services.

The study also revealed that although the availability of PHC services within FHCs was close to 100%, service specific readiness varied from 44.0 to 83.6%. Readiness among FHCs to provide specific services were hampered, mostly due to the unavailability of essential medicines and diagnostic tests. It should be noted that there are clear inconsistencies between clinical guidelines [13–17] and government regulations [23]. If the former requires FHC doctors to treat patients and list medicines, the latter does not even specify that FHCs should have medicines in stock. The FHC standards [24] and package of essential services [8] stipulate that FHCs should provide emergency care, daycare, nursing, palliative care etc.; however, there is no system in place for procuring and supplying medicines and commodities for FHCs in Mongolia. Furthermore, family planning, HIV, and TB services highly rely upon support from external donors. Government policies regarding free-of-charge PHC [25], and the use of holistic medical approaches when delivering PHC services (with a comprehensive set of preventive, diagnostic, treatment, and referral activities) [26], have yet to be translated into practical implementations.

Unavailability of diagnostic and treatment services within FHCs force people to refer to district or tertiary level hospitals (or even private hospitals); hence, system inefficiencies arise. Self-referrals and high rates of inappropriate admissions within district and tertiary level hospitals are well documented [27, 28]. The unavailability of medicines within FHCs also contributes to increased direct payments, as people need to shoulder the cost of medicines. The national health insurance fund subsidizes the cost of around 300 essential medicines for insured people; however, access to these medicines is highly limited, and the listed items are becoming non-essential [29]. According to a national household survey in 2014, 69.0% of households’ out-of-pocket spending on health went toward medicines [30]. The WHO estimated that 95.0% of out-of-pocket expenditures occurred owing to medicinal purchases in 2011 [31]. Overall out-of-pocket costs accounted for 39.0% of total health expenditures in 2015, and 1.1% (or 20,000 people) were forced into poverty due to healthcare costs every year [32, 33].

A major factor underlying low service readiness among FHCs is a lack of PHC funding, which has been a problem in Mongolia since the inception of FHCs [34–36]. The amount paid under a capitation fee ($4.5 per person per year) is too low to cover costs related to primary care and services that FHCs are supposed to provide. FGP/FHCs have never been prioritized in terms of resource allocation. While government allocation for PHC has increased over the past 15 years (from 17.5% of public health expenditures in 2000 to 25.0% in 2016), the proportion of government spending on FHCs has remained at around 4.0% during this period. It should be noted that the share of total government expenditures in terms of GDP has decreased from 4.6% in 2000 to 2.8% in 2016, two times lower than what the WHO recommends (5.0%) [37].

The present study also demonstrated that despite a high density of health facilities in the two districts, only FHCs offered PHC. It should also be noted that FHC services are mostly utilized by the poor and vulnerable individuals who rely on free services [38–40]. If shortcomings in PHC provisions are not addressed, an inefficient and low-performing system will deepen the pro-poor inequality in FHC service utilization. We also argue for the role of private clinics and sanatoriums in providing health services. The contribution of these entities in influencing overall health system performance should be evaluated.

A few study limitations should be noted. For instance, the present findings may not be fully generalizable to other health facilities in Mongolia. Our study only covered two districts in Ulaanbaatar, accounting for only 25% of the total citywide population. However, this was the first attempt to assess, using the SARA, the level of accountability among health facilities in terms of healthcare output. Assessing health “entities” is one of five key aspects (entity, output, input, external influences, and links with the rest of the health system) that facilitate better understanding and interpretation of healthcare efficiency and performance [41]. Therefore, we argue for documenting various assessment findings. Further studies that cover more districts, especially rural provinces, are also required. Additionally, linking health facility assessments with household surveys regarding actual service utilization should be considered in future studies.

## Conclusions

Free and universal PHC is stipulated within various policies and regulations in Mongolia; however, the present results revealed that availability of basic health services within specific facilities is insufficient. Among all facility types, FHCs contribute most to PHC provisions, but readiness was mostly hampered by a severe lack of diagnostic capacities and essential medicines. Declaring free access does not mean ensuring access. Policies need to be translated into tangible, comprehensive, coordinated, and forceful actions to address capacity limitations. If provisional shortcomings among FHCs in Ulaanbaatar are not addressed appropriately, the current system will further contribute to overall health inefficiencies, financial inequalities, and insecurities.

The present study was the first to assess PHC availability at different health facility entities in Ulaanbaatar, as well as investigate readiness to provide PHC using the SARA method. Although this tool does not reflect other dimensions of access (such as geographic or financial barriers), affordability, and service utilization patterns, the present results help with judging how well the current health system is accomplishing its stated goals.

## Abbreviations

ADB: Asian Development Bank
CRD: chronic respiratory disease
CVD: cardiovascular disease
FGP: family group practice
FHC: family health center
GRS: general readiness score
HIV: human immunodeficiency virus
NCD: non-communicable disease
PHC: primary health care
SARA: service availability and readiness assessment
SSAS: specific service availability score
SSRS: specific service readiness score
STI: sexual transmitted disease
TB: tuberculosis
WHO: World Health Organization
